# Interferon-alpha Subtype 11 Activates NK Cells and Enables Control of Retroviral Infection

**DOI:** 10.1371/journal.ppat.1002868

**Published:** 2012-08-09

**Authors:** Kathrin Gibbert, Jara J. Joedicke, Andreas Meryk, Mirko Trilling, Sandra Francois, Janine Duppach, Anke Kraft, Karl S. Lang, Ulf Dittmer

**Affiliations:** 1 Institute for Virology of the University Hospital in Essen, University of Duisburg-Essen, Essen, Germany; 2 Institute for Immunology of the University Hospital in Essen, University of Duisburg-Essen, Essen, Germany; National Institutes of Health/National Institute of Allergy and Infectious Diseases, United States of America

## Abstract

The innate immune response mediated by cells such as natural killer (NK) cells is critical for the rapid containment of virus replication and spread during acute infection. Here, we show that subtype 11 of the type I interferon (IFN) family greatly potentiates the antiviral activity of NK cells during retroviral infection. Treatment of mice with IFN-α11 during Friend retrovirus infection (FV) significantly reduced viral loads and resulted in long-term protection from virus-induced leukemia. The effect of IFN-α11 on NK cells was direct and signaled through the type I IFN receptor. Furthermore, IFN-α11-mediated activation of NK cells enabled cytolytic killing of FV-infected target cells via the exocytosis pathway. Depletion and adoptive transfer experiments illustrated that NK cells played a major role in successful IFN-α11 therapy. Additional experiments with Mouse Cytomegalovirus infections demonstrated that the therapeutic effect of IFN-α11 is not restricted to retroviruses. The type I IFN subtypes 2 and 5, which bind the same receptor as IFN-α11, did not elicit similar antiviral effects. These results demonstrate a unique and subtype-specific activation of NK cells by IFN-α11.

## Author Summary

The innate immune response mediated by cells such as natural killer (NK) cells can contribute to immunity against viral infections. NK cells can kill virus-infected cells and thus inhibit virus replication and spread during acute infection. However, in infections with retroviruses, like HIV, these cells are not sufficient to prevent pathology. Here, we describe a new strategy to augment natural killer cell responses during virus infections by using a subtype of the type I interferon family as antiviral drug. This therapy strongly activated NK cells and enabled them to control retrovirus as well as herpes virus infections in mice. The new approach might have great potential for the treatment of many infectious and tumor diseases in which natural killer cells play a significant role in immunity.

## Introduction

Natural killer (NK) cells play an important role in the host defense against viral infections. They can recognize and kill virus-infected cells and produce cytokines like Interferon-γ (IFN), which enhance innate and adaptive immunity [1]. In contrast to B and T lymphocytes, NK cells do not rearrange their receptors somatically but they express various activating and inhibitory receptors on their surfaces, which recognize major histocompatibility complexes (MHC), as well as other ligands [2].

NK cells were shown to be critical for the control of herpes virus infections, like herpes simplex virus-1 or cytomegalovirus (CMV) [3], [4]. There is also some evidence emphasizing their importance in retroviral infections. Polymorphisms in Killer-cell immunoglobulin-like receptors and human leukocyte antigen (HLA), which significantly delay AIDS-progression in HIV-infected individuals, have been described [5]. Three single nucleotide polymorphisms located in the HLA gene locus were shown to be associated with lower viral set points in chronic HIV-1 patients and thus an influence on NK cell recognition was proposed [6]. Furthermore, immune escape from NK cell responses has been described for HIV-1. For example, the ligands for the activating NK cell receptor NKG2D (MICA, ULBP1 or ULBP2) are down-regulated by the HIV Nef protein on infected cells, which might protect them from NK cell killing [7]. Until now, it is still not fully understood how NK cells recognize HIV-infected cells and how much they can contribute to HIV control, but it is quite obvious that they cannot completely prevent HIV pathology. In other retroviral infections, like Friend Virus (FV) infection of mice, NK-cell responses have also been described [8]. However, depletion experiments showed that they do not contribute significantly to the control of acute FV replication [9]. Thus, augmenting NK cell responses during acute retroviral infection might be a promising therapeutic approach to enhance retroviral immunity. It was shown that cytokines like Interleukin (IL)-12, IL-15, IL-18 or type I IFN are required for efficient NK cell activation. Conventional dendritic cells (DC) produce the cytokines IL-12, IL-15 and IL-18 that activate NK cells [10], whereas plasmacytoid DC produce large amounts of type I IFN, which is required for NK cell proliferation and cytotoxicity [11]. During HIV infection, the initial type I IFN response is rapid but only very transient [12] and during FV infection no type I IFN is detectable in the plasma of infected mice [13], [14]. This weak type I IFN response during retroviral infections might contribute to an inefficient NK cell activation and incomplete NK cell-mediated control of virus replication. Thus, therapeutic improvement of NK cell responses using exogenous IFN-α might be a good strategy to treat retroviral infections. An activating effect of IFN-α on NK cells was previously described in HCV-infected patients treated with IFN-α2a. The treatment increased the cytotoxicity of NK cells, and this correlated with reduced viral loads [15], [16], but in human, it is difficult to define the *in vivo* contribution of NK cells to the antiviral response since experiments such as NK depletions or transfers are not possible. In addition, very little is known about the antiviral effect of type I IFN and NK cells in retroviral infections.

Therefore, we used the FV mouse model to develop a new strategy to therapeutically improve NK cell responses with IFN-α subtypes during acute retroviral infection. FV is a retroviral complex (Friend murine leukemia virus (F-MuLV) and Spleen focus-forming virus (SFFV)) that induces acute splenomegaly in susceptible mice due to a rapid polyclonal erythroblast proliferation, which is followed by the development of a lethal erythroleukemia [17]. Infected mice were treated with exogenous type I IFN during acute FV infection and antiviral NK cell responses were determined.

Type I IFN belong to a multigene family consisting of several IFN-α subtypes but only one IFNβ [18]. All type I IFN subtypes are genetically highly conserved [19]. Interestingly, they all bind the same ubiquitously expressed receptor (IFN-α/β receptor, IFNAR) but they differ in their biological activities [20]. Hints to explain the distinct functions come from studies reporting that various human IFN-α subtypes bind with different affinities to the 2 receptor subunits [21]. This leads to distinct downstream signaling cascades shown by phosphorylation of STAT molecules and MAP kinases [22] and finally results in unique expression patterns of IFN-stimulated genes (ISG) for each IFN-α subtype [23]. This is in line with results from *in vitro* and *in vivo* studies on several viral infections that report distinct antiviral activities of specific IFN-α subtypes [18], [24]–[27]. However, these studies do not address the antiviral mechanisms of IFN-α subtypes, which most likely include important immunomodulatory properties. Here, we determined the stimulatory effect of 3 different IFN-α subtypes on NK cell responses and their ability to reduce FV infection *in vivo*. Only IFN-α11 showed a potent antiretroviral effect and selectively augmented NK cell responses during acute infection. Such defined immunotherapies with specific IFN-α subtypes should be interesting new strategies to treat viral infections or cancer.

## Materials and Methods

### Ethics statement

Animal experiments were performed in strict accordance with the German regulations of the Society for Laboratory Animal Science (GV-SOLAS) and the European Health Law of the Federation of Laboratory Animal Science Associations (FELASA). The protocol was approved by the North Rhine-Westphalia State Agency for Nature, Environment and Consumer Protection (LANUF) (Permit Number: G1204/11). All efforts were made to minimize suffering.

### Mice and virus

Three to 6 month old female (B10.A×A.BY) F_1_ mice (H-2^a/b^) (Jackson, USA) and C57BL/6 mice (H-2^b/b^) (Harlan, Germany) were used for the experiments. Experiments were also done with congenic CD45.1^+^ C57BL/6 mice, IFNAR^−/−^ mice [28] and GzmB^−/−^ [29] mice backcrossed more than 10 times on C57BL/6 background. The B-tropic, polycythemia-inducing FV complex used in all experiments was taken from uncloned virus stocks obtained from 15% spleen cell homogenates from BALB/c mice infected 14 days previously with 3,000 spleen focus-forming units (SFFU) [30]. The used stock was not contaminated by lactate dehydrogenase-elevating virus. The progression of disease was monitored by spleen weights and virus assays as indicated.

In all experiments, (B10.A×A.BY) F_1_ mice were injected intravenously with 0.5 ml PBS containing 7000 SFFU of the FV-complex. C57BL/6, GzmB^−/−^, CD45.1^+^ and IFNAR^−/−^ mice were injected intravenously with 0.5 ml PBS containing 20,000 SFFU of the FV complex. MCMV, strain Smith, a DNA virus of the herpes virus family, was obtained from salivary glands of infected BALB/c mice [31]. For acute virus infections, C57BL/6 mice were injected intraperitoneally with 4×10^5^ plaque forming units (PFU) of MCMV.

### Expression of IFN-α subtypes and measurement of IFN-α subtype concentrations

Expression of IFN-α2 and IFN-α5 were performed as previously described [32]. To produce murine IFN-α11, the cell line HEK293mIFNalpha11 was cultivated as described [33]. All concentrated supernatants were tested for IFN-α subtype concentration by a virus-free, cell-based bioassay using Mx/Rage 7 cells [32], [33]_ENREF_18_ENREF_17.

### IFN-α inhibition assay

*Mus dunni* tail fibroblast cells were pre-treated *in vitro* for 24 h with increasing concentrations (2.5–40 units/ml) of IFN-α2, α5 or α11. Cells were then infected with 25 FFU of F-MuLV, cultivated for 3 days, fixed with ethanol, stained with F-MuLV envelope-specific mAb 720, and developed with peroxidase-conjugated goat anti-mouse antibody and aminoethylcarbazol to detect foci [34].

### IFN-α subtype treatment

Mice were injected intraperitoneally daily from day −1 through +9 or day +3 through +9 of infection with 8000 units of the different IFN-α subtypes. Control mice were injected with the supernatant of 293T cells transfected with an empty vector. One hour post injection, similar IFN-α concentrations were measured for all different IFN-α subtypes in the blood (data not shown). Ten days post infection, the mice were sacrificed and analyzed for disease progression and viral loads.

### Detection of virus-infected cells

Infectious center (IC) assays were performed as described previously [34]. The number of MCMV PFU was determined by plaque assay using a 10% homogenate of tissue taken from individual mice and tenfold dilutions of this homogenate on GT-KO cells were used to quantify MCMV titers [35]. Titers reported are numbers of log_10_ PFU per whole spleen, liver and salivary glands.

### RNA isolation

Total RNA was isolated from splenocytes utilizing TRIzol Reagent (Life technologies) and NucleoSpin RNA II (Macherey-Nagel). Isolated RNA was dissolved in RNase-free water and stored at −80°C.

### Real-time-PCR

Real-time-PCR (RT-PCR) analysis for the quantification of *OAS1a* and *PKR* mRNA was performed using QIAGEN One Step RT-PCR kit and QIAGEN Quanti Tect Primer assay for *OAS1a* and *PKR*. The quantitative mRNA levels were performed by using StepOne Software v2.2 (Applied Biosystems) and were normalized to β-actin mRNA expression levels.

### Tetramers and tetramer staining

For quantification of virus-specific CD8^+^ T cells, spleen cells were stained with anti-CD8 (Ly-2) and MHC class I H2-D^b^ tetramers specific for the FV GagL epitope [36] for 30 min at room temperature. For detection of virus-specific CD4^+^ T cells, spleen cells were stained with MHC class II-antibody tetramers specific for F-MuLV env fn20 [37] for 3 h at 37°C. Subsequently, cells were stained with anti-CD4 (RM4–5) for additional 30 min at 37°C. Cells were washed twice, resuspended in buffer containing 7-aminoactinomycin D (7AAD) and analyzed by flow cytometry.

### Cell-surface and intracellular staining

Cell-surface staining was performed with the following antibodies: anti-CD3 (17A2), anti-CD49b (DX5), anti-CD69 (H1.2F3), anti-NK1.1 (PK136) and anti-TRAIL (N2B2). Dead cells (positive for fixable viable dye; eBioscience) were excluded from analysis. Intracellular granzyme B (clone GB12) staining was performed as described [38]. Data were acquired on a LSR II flow cytometer (BD Biosciences) and analyses were performed using FACSDiva (BD Biosciences) and Flow Jo (Tree Star) software.

### *In vivo* cytotoxicity assay

The *in vivo* cytotoxicity assay was performed as previously described [39].

### NK cell depletion

Mice were injected intraperitoneally with 0.5 ml of supernatant fluid containing NK1.1-specific monoclonal antibody PK136. Mice were injected every other day starting on the day of FV or MCMV infection.

### NK cell cytotoxicity assay *in vitro*

*In vitro* cytotoxicity assay was performed using the lactate dehydrogenase release (LDH) assay (CytoTox 96 nonradioactive cytotoxicity assay, Promega) according to the manufacturer's protocol. NK cells were isolated from splenocytes of naive mice, FV-infected and FV-infected IFN-α11-treated mice at 10 days post infection (dpi) by MACS technology (Miltenyi Biotec). The cytotoxicity assay was performed in 96-well U-bottom plates with 1×10^4^ YAC1 [40] or FBL-3 cells [41] with different numbers of NK cells to obtain the effector to target ratios as indicated. Absorbance was recorded at 490 nm using an Expert Plus Microplate Reader (Biochrom). In some experiments, the isolated NK cells were pretreated with 20 nM concanamycin A (CMA) (Sigma-Aldrich) for 2 h to inactivate perforin [42]. Caspase-dependent cytotoxicity was evaluated by adding 100 µM Z-VAD-fmk (Peptanova) to the cells. TRAIL- and Fas-mediated cytotoxicity was analyzed by adding mouse neutralizing anti-TRAIL (10 µg/ml; clone N2B2), anti-Fas ligand (10 µg/ml; clone MFL3) or isotype control (10 µg/ml).

### Mixed bone marrow chimeras

Bone marrow cells were collected from the femurs and tibias of CD45.1^+^ wild type or CD45.2^+^ IFNAR^−/−^ mice. C57BL/6 recipient mice were irradiated at 1050 rad and reconstituted 24 h later with a mixture of bone marrow cells composed of 5×10^6^ cells from CD45.1^+^ wild type and 5×10^6^ cells from CD45.2^+^ IFNAR^−/−^ mice. Thirty days later, mice were treated with IFN-α11 and infected with FV according to the standard protocol.

### Adoptive transfer of NK cells

NK cells were isolated from splenocytes of FV-infected and FV-infected IFN-α11-treated mice at 9 dpi by MACS technology (Miltenyi Biotec). Adoptive transfers were done by i.v. injection of 2×10^6^ cells in PBS. Recipient mice were infected with FV and received NK cells at 6 dpi. Four days post transfer, the spleen samples were assayed for viral loads.

### Statistical analyses

Statistical analyses and graphical presentations were computed with Graph Pad Prism version 5. Statistical differences between 2 different groups were analyzed by the unpaired student's t test. Analyses including several groups were tested using the Kruskal-Wallis one-way analysis of variance on ranks and Dunn's multiple comparison test.

## Results

### Antiretroviral activity of IFN-α11 *in vivo*

**Figure 1:**
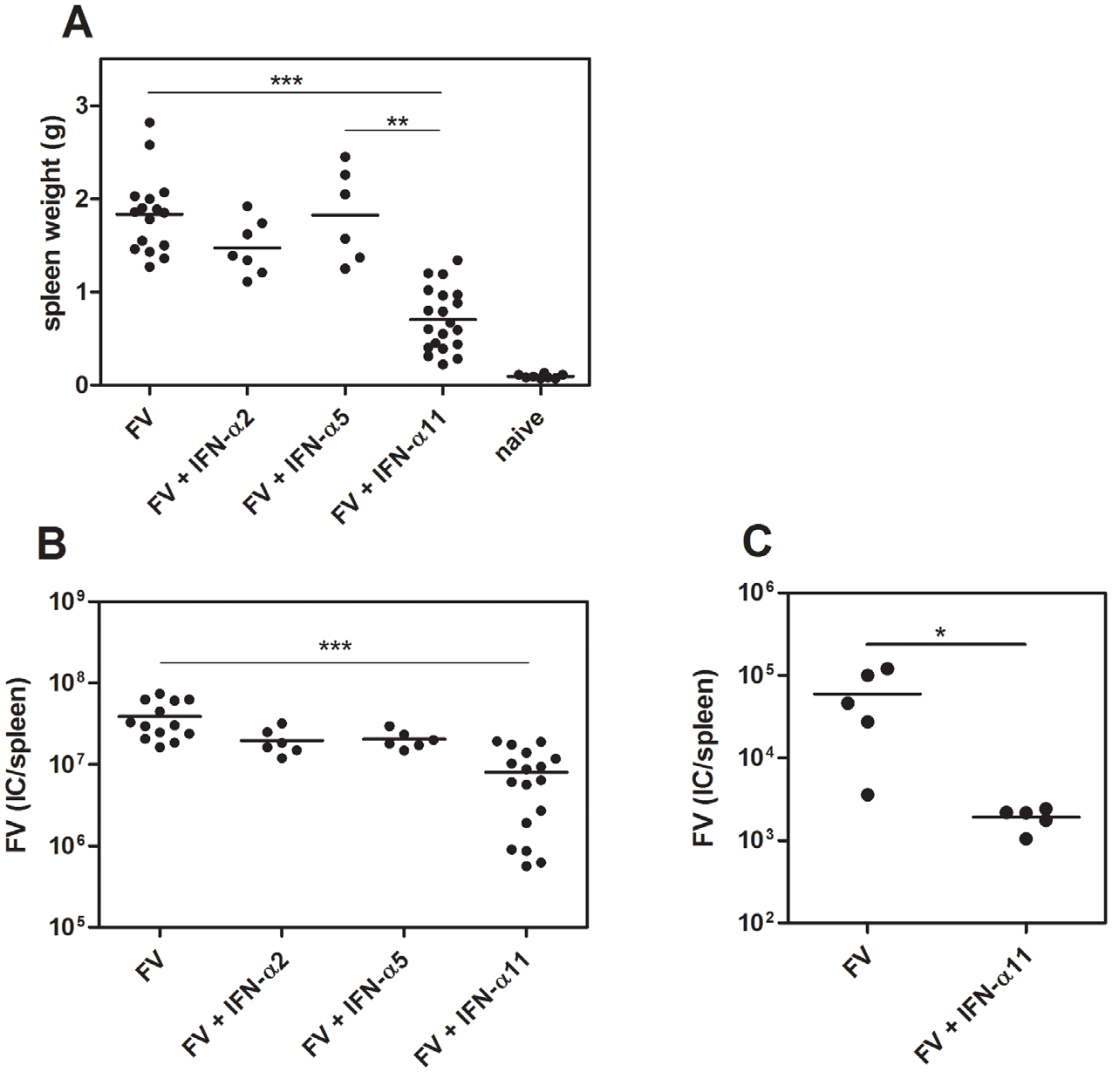
Antiretroviral activity of different IFN-α subtypes *in vivo*. (B10.A×A.BY) F1 mice were treated daily with 8000 units of IFN-α2, α5 or α11 from −1 through +9 dpi with 7000 SFFU of FV. Ten dpi, disease progression and viral loads were analyzed. Virus-induced splenomegaly was documented by spleen weights (A) and viral loads were measured in the spleen (B) by using an infectious center assay. To analyze the long-term protection of IFN-α11 treatment, we treated mice with IFN-α11 during the acute phase of FV infection (−1 through +9 dpi). At 6 weeks post infection, viral loads were analyzed by an infectious center assay (C). At least 5 mice per group were analyzed and the mean values for each group are indicated by a bar. At least 2 independent experiments were performed. Differences between the control group (FV) and the groups of treated mice (FV+IFN-α2, α5, or α11) were analyzed.

Since universal IFN-α, a mixture of several different IFN-α subtypes, has a strong inhibitory effect on FV replication [14], we analyzed the potency of the 3 subtypes IFN-α2, α5 and α11 to inhibit and control FV replication *in vivo*. Therefore, we infected susceptible (B10.A×A.BY) F_1_ mice with FV and treated them daily with 8000 units of the different IFN-α subtypes starting one day prior to infection. At 10 dpi, mice were analyzed for viral loads and FV-induced splenomegaly. The IFN-α11-treated mice had 2.6-fold lighter spleens (mean: 0.7 g) compared to FV-infected control mice (mean: 1.8 g) (Fig. 1A). In contrast, the application of IFN-α2 or α5 did not significantly reduce spleen weights (Fig. 1A). Similarly, spleen viral loads were significantly reduced by IFN-α11 treatment (mean viral loads per spleen: 7.9×10^6^) compared to untreated control mice (mean viral loads per spleen: 3.8×10^7^), whereas IFN-α2 or α5 did not reduce viral loads (Fig. 1B). Also treatment with very high concentrations of IFN-α2 or α5 (40,000 units of IFN-α2 or 32,000 units of IFN-α5) did not significantly reduce viral loads indicating that the lack of activity cannot be overcome by increasing concentrations (data not shown). Therapeutic post-exposure treatment with IFN-α11 starting at 3 dpi showed similar results with significantly reduced splenomegaly and viral loads (data not shown). Long-term experiments also demonstrated that IFN-α11 treatment during acute FV infection resulted in a sustained reduction in viral loads and prevented the development of lethal erythroleukemia (Fig. 1C), demonstrating the high antiviral potential of IFN-α11.

### Activated NK cells played a crucial role in successful IFN-α11 therapy

**Figure 2:**
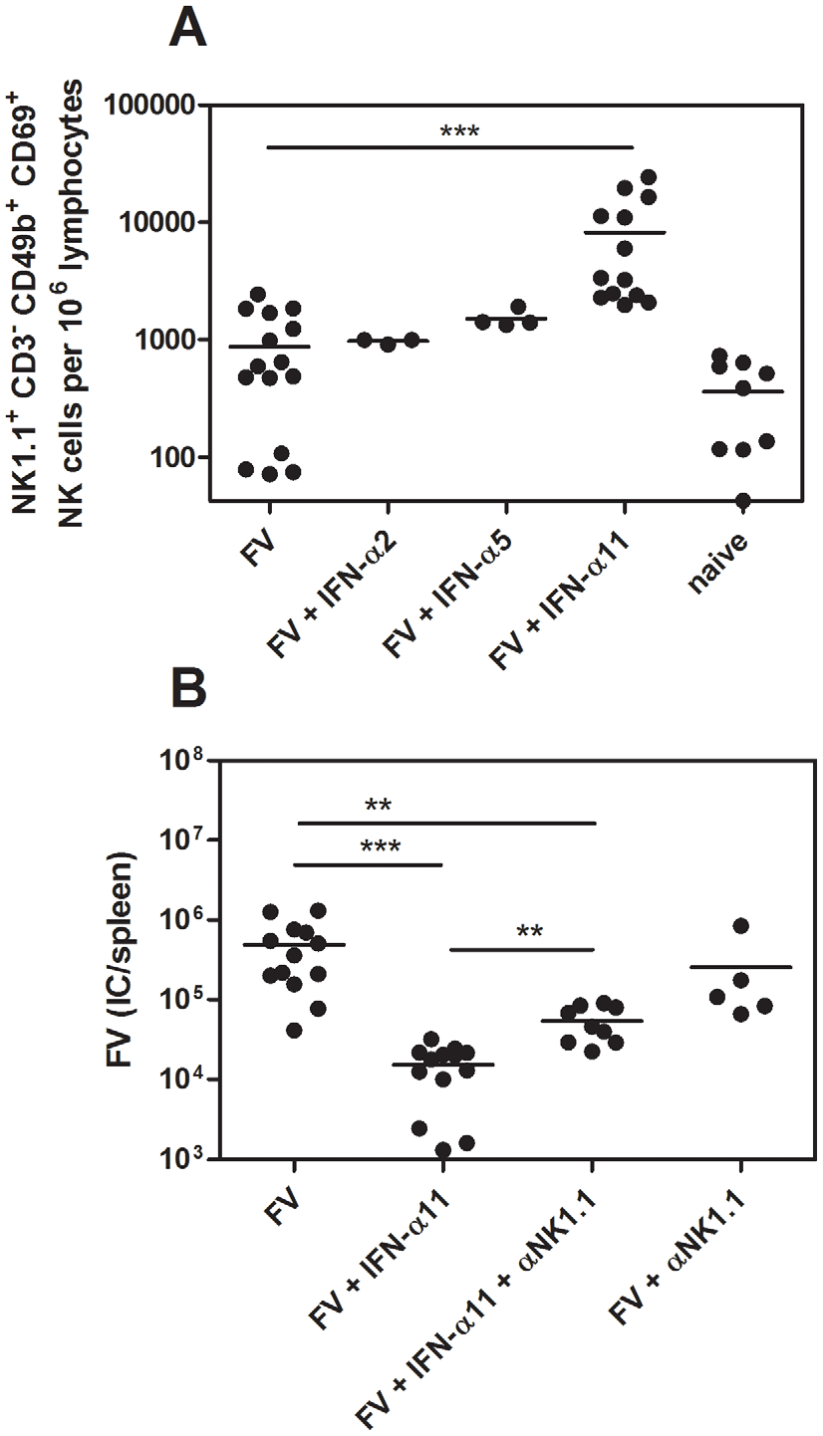
Analysis of NK cells from IFN-α11-treated mice. (B10.A×A.BY) F_1_ mice were treated daily with 8000 units of IFN-α2, α5 or α11 from −1 through +9 dpi with 7000 SFFU of FV. At 10 dpi, splenocytes were analyzed by flow cytometry. The early activation marker CD69 was used to analyze the frequencies of activated NK cells (NK1.1^+^ CD3^−^ CD49b^+^) (A). C57BL/6 mice were treated daily with 8000 units of IFN-α11 from −1 through +9 dpi with 20,000 SFFU of FV. NK cells were depleted by 5 injections of supernatant of clone PK136, starting at the day of infection. IFN-α11-treated non-depleted mice and NK cell depleted, non-treated mice were used as control groups. At 10 dpi viral loads in the spleen were determined (B). A minimum of 3 mice per group were analyzed and the mean values are shown by bars. At least 2 independent experiments were performed. Statistically significant differences between the groups are indicated by ** for p<0.005, *** for p<0.0005.

NK cells are able to kill virus-infected target cells and thus restrict viral replication. Hence, we investigated whether the antiretroviral response, which was observed *in vivo* (Fig. 1), was mediated by NK cells. First, we analyzed IFN-α2, α5 and α11-treated and untreated FV-infected mice at 10 dpi for the activation status of their splenic NK cells (Fig. 1). As shown in Fig. 2A, the frequency of activated NK cells (CD69^+^) was significantly increased after IFN-α11 treatment (mean: 8200 NK1.1^+^CD3^−^CD49b^+^CD69^+^ cells) compared to FV-infected control mice (mean: 930 NK1.1^+^CD3^−^CD49b^+^CD69^+^ cells). This effect was highly specific for IFN-α11, as α5 or α2 treatment did not influence the NK cell activation (Fig. 2A). The data suggest that NK cells might play an important role in the elimination of FV-infected cells after IFN-α11 treatment. To test this hypothesis, we performed NK cell depletions during IFN-α11 therapy of acute FV infection. For these experiments and some of the following experiments, C57BL/6 mice instead of the more susceptible (B10.A×A.BY) F1 mice were used because NK depletion is more efficient in this strain, and knockout mice were needed for some of the experiments. Note that the viral loads in C57BL/6 mice were lower than in (B10.A×A.BY) F1 mice (Fig. 1B and 2B) but the therapeutic effect of IFN-α11 was also highly significant in C57BL/6 mice (Fig. 2B). At 10 dpi, ≥99.5% of the splenic NK cells (NK1.1^+^CD3^−^CD49b^+^) from C57BL/6 mice were ablated by the injection of NK1.1-specific antibodies (data not shown). The NK cell depletion during IFN-α11 therapy resulted in a 4.3-fold increase in mean viral loads compared to non-depleted IFN-α11-treated mice (Fig. 2B). However, NK cell depletion abolished only part of the antiretroviral effect of IFN-α11, and the mean viral load in the IFN-α11-treated, NK cell-depleted group was still significantly lower than in the control group of FV-infected mice. The data indicate that activated NK cells partially contributed to the antiretroviral effect of IFN-α11. In contrast, virus-specific CD4^+^ T cell responses were not significantly augmented by IFN-α11 (mean: 1900 CD4^+^ tetramer-II^+^ T cells) compared to FV-infected control mice (mean: 910 CD4^+^ tetramer-II^+^ T cells) (Fig. S2), whereas an enhanced frequency of virus-specific CD8^+^ T cells was observed after IFN-α11 treatment. (mean: 3700 CD8^+^ tetramer-I^+^ T cells) compared to FV-infected control mice (mean: 900 CD8^+^ tetramer-I^+^ T cells). However, the therapy with IFN-α11 significantly decreased cytotoxic activity of the FV-specific CD8^+^ T cells *in vivo* (Fig. S2C) excluding their contribution to the therapeutic effect.

Thus, a more direct antiviral activity of IFN-α11 may account for the other part of its antiviral effect. Type I IFN can induce the expression of enzymes, like proteinkinase R (PKR) or oligoadenylate synthetase (OAS1a), that directly inhibit viral transcription or protein translation. To investigate if treatment with IFN-α11 induced these enzymes, we analyzed the induction of *PKR* and *OAS1a* in splenocytes of FV-infected, IFN-α11-treated mice *in vivo* (Fig. S1B). The mRNA levels of both antiviral enzymes were significantly increased in IFN-α11-treated mice compared to untreated FV-infected controls (Fig. S1B). The antiretroviral activity of all 3 IFN-α subtypes was also investigated in a NK cell-free cell culture system using different IFN concentrations (Fig. S1A). IFN-α11 was very potent in inhibiting F-MuLV replication *in vitro* (inhibition: 79.4% at the highest IFN-α concentration). IFN-α2 could not inhibit F-MuLV replication *in vitro* and IFN-α5 blocked F-MuLV replication only at a very high concentration (Fig. S1A). These results highlight that IFN-α11 induced ISG with direct antiviral potential likely contributed to its anti-FV activity *in vivo*.

### IFN-α11 treatment improved NK cell effector functions

**Figure 3:**
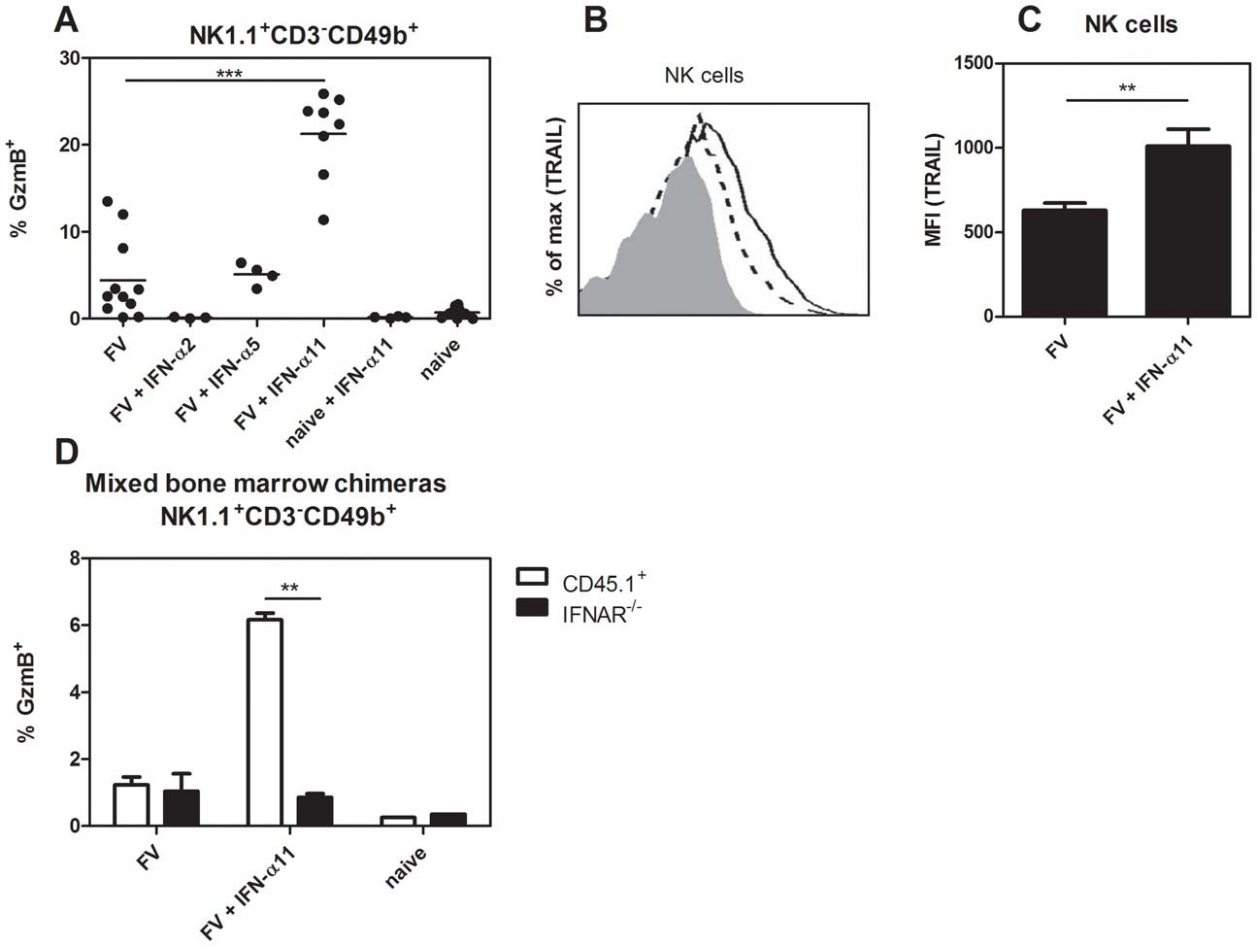
Analysis of NK cell effector molecules in IFN-α11-treated mice. (B10.A×A.BY) F_1_ mice were treated daily with 8000 units of IFN-α2, α5 or α11 from −1 through +9 dpi with 7000 SFFU of FV. At 10 dpi, splenocytes were analyzed by flow cytometry. The frequencies of intracellular expression of granzyme B (GzmB) in NK cells (NK1.1^+^ CD3^−^ CD49b^+^) are shown (A). Surface TRAIL expression was measured in the same NK cell population of the IFN-α11-treated mice (shaded histogram = isotype control; dashed line = FV; solid line = FV+IFN-α11) (B). The mean fluorescence intensity of TRAIL expression was evaluated on NK cells (C). A minimum of 3 mice per group were analyzed and the mean values are shown by bars. To analyze whether IFN-α11 acts directly on NK cells, C57BL/6 mice were irradiated and reconstituted with mixed bone marrow cells from CD45.1^+^ wild type and CD45.2^+^ IFNAR^−/−^ mice at a ratio of 1∶1. Thirty days later, mice were treated daily with 8000 units of IFN-α11 from −1 through +9 dpi with 20,000 SFFU of FV. As controls, mixed bone marrow chimeras were infected with FV or stayed naive. At 10 dpi, splenocytes were analyzed by flow cytometry. The frequencies of intracellular expression of granzyme B in CD45.1^+^ wild type (white bars) and CD45.2^+^ IFNAR^−/−^ (black bars) NK cells are shown (D). Eight mice in each group of infected mice were analyzed and the mean value for each group is indicated by a bar. At least 2 independent experiments were performed. Statistically significant differences between the control group (FV) and the IFN-α11-treated mice are indicated by ** for p<0.005 or *** for p<0.0005.

The NK depletion experiments suggested an important antiviral effect of NK cells during IFN-α11 treatment. NK cells mediate their effector function through the recognition and killing of virus-infected cells by releasing cytotoxic granules containing e.g. granzyme B and perforin, or the expression of ligands for death receptors, e.g. TRAIL or FasL. We sacrificed IFN-α subtype-treated and non-treated FV-infected mice at 10 dpi and analyzed splenic NK cells for the expression of granzyme B (Fig. 3A) or TRAIL (Fig. 3B+C). A sixfold increase in the percentages of NK cells expressing granzyme B was observed in IFN-α11-treated mice (mean: 21% GzmB^+^ of NK1.1^+^CD3^−^CD49b^+^) compared to FV-infected control mice (mean: 6%). IFN-α11 treatment also significantly up-regulated the surface expression of TRAIL on splenic NK cells compared to untreated controls as shown in a representative flow cytometry histogram and for mean fluorescence intensities of TRAIL expression on NK cells (Fig. 3B and C). In contrast, IFN-α2 or α5 treatment did not increase the expression of cytotoxic molecules by NK cells (Fig. 3A). To gain full effector functions, NK cells have to be primed by different cytokines like type I IFN, IL-12, IL-18 or IL-15_ENREF_34. Thus, we investigated whether IFN-α11 activates NK cells directly or if the priming is mediated indirectly by other cells. We irradiated C57BL/6 mice and 24 h later we injected bone marrow cells intravenously from IFN-α/β receptor knockout mice (IFNAR^−/−^, CD45.2^+^) and wild type mice (CD45.1^+^) (ratio 1∶1). Thirty days post cell transfer we infected these mice with FV and treated them with IFN-α11 according to the standard protocol. Ten dpi, the mice were analyzed for granzyme B expression in IFNAR^−/−^ NK cells (CD45.2^+^) versus wild type NK cells (CD45.1^+^). More than 5% of all CD45.1^+^ wild type NK cells expressed granzyme B after IFN-α11 treatment, whereas only 0.8% of the CD45.2^+^ IFNAR^−/−^ NK cells produced granzyme B post treatment (Fig. 3D). This suggests that IFN-α11 acted directly on NK cells via binding to the IFNAR.

### IFN-α11 induced granzyme B-dependent killing of virus-infected target cells

**Figure 4:**
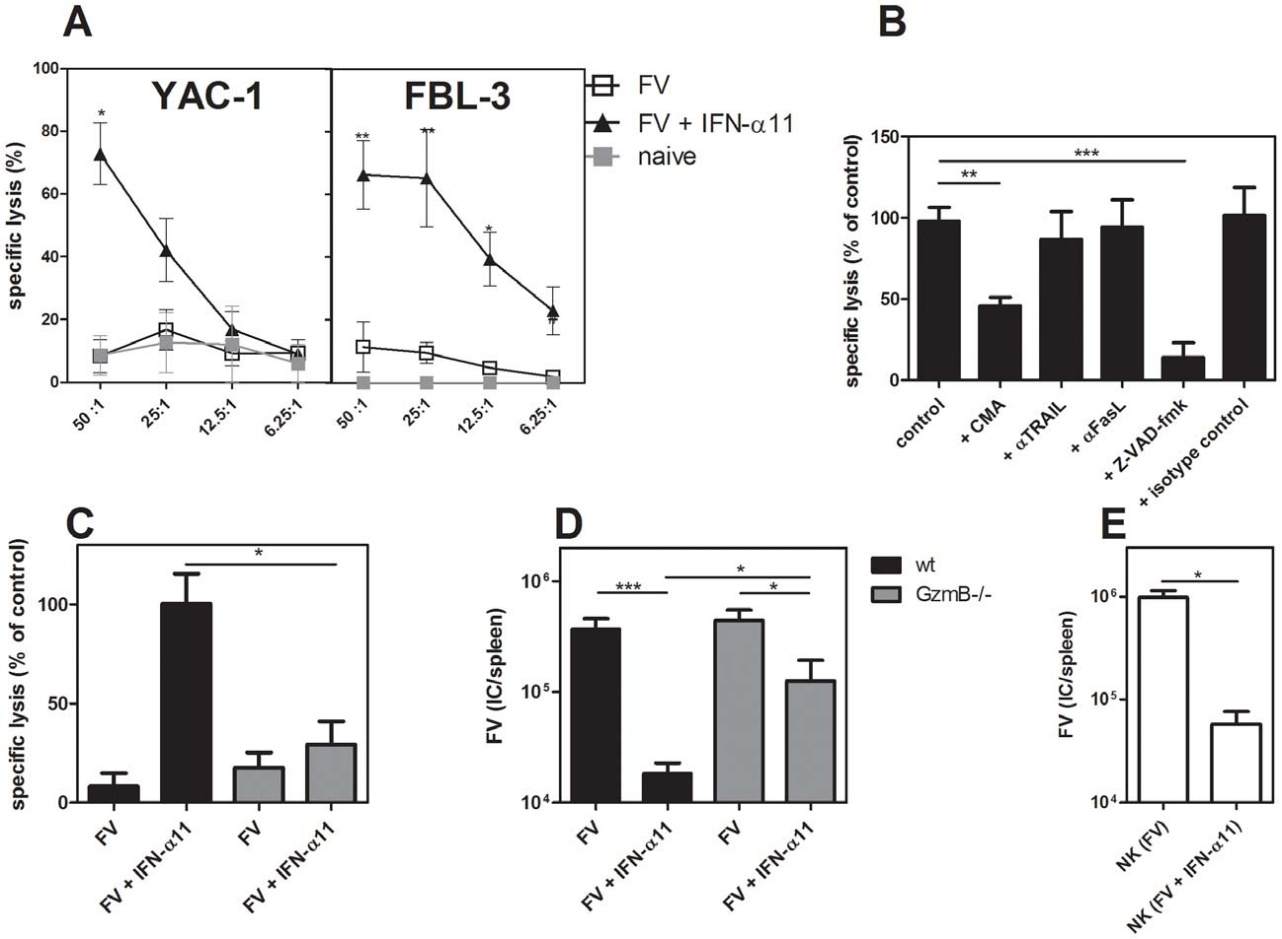
Target cell killing by NK cells from IFN-α11-treated mice. C57BL/6 mice were treated daily with 8000 units of IFN-α11 from −1 through +9 dpi with 20,000 SFFU of FV. Ten dpi, NK cells were isolated from 10 individual mice per group using magnetic bead separation. NK cells were incubated for 4 hours with 2 different target cell lines (YAC-1 and FBL-3) in different effector to target cell (E∶T) ratios (A). In some assays, CMA, Z-VAD-fmk, neutralizing anti-TRAIL, anti-Fas ligand (FasL) mAbs, or isotype control antibodies were added to NK and FBL-3 cells (E∶T = 25∶1) (B). Killing of FBL-3 cells by NK cells (E∶T = 25∶1) isolated from mice deficient in granzyme B (GzmB^−/−^; grey bars) and wild type mice (C57BL/6; black bars) was investigated (C). Mice were treated daily with 8000 units of IFN-α11 from −1 through +9 dpi with 20,000 SFFU of FV. Ten dpi, NK cells were isolated from six individual mice per group using magnetic bead separation. Specific lysis of target cells was measured using the CytoTox 96 Non-Radioactive Cytotoxicity Assay. Mice deficient in granzyme B (GzmB^−/−^; grey bars) and wild type mice (C57BL/6; black bars) treated with IFN-α11 or left untreated were analyzed at day 10 post FV infection for viral loads in the spleen by using an infectious center assay (D). Adoptive transfer experiments of NK cells were performed as follows. C57BL/6 mice were treated daily with 8000 units of IFN-α11 from −1 through +9 dpi with 20,000 SFFU of FV or left untreated. Nine dpi, NK cells were isolated from 10 individual mice per group. 2×10^6^ NK cells were adoptively transferred into recipient mice, which were infected with FV 6 days before transfer. Four days post transfer, spleen samples were assayed for viral loads by using an infectious center assay (E). Five mice in each group of infected mice were analyzed and the mean value for each group is indicated by a bar. At least 2 independent experiments were performed. Statistically significant differences between the groups are indicated by * for p<0.05, ** for p<0.005 or *** for p<0.0005.

We demonstrated an increased expression of granzyme B and TRAIL in splenic NK cells after IFN-α11 treatment. To verify that this up-regulation of cytotoxic molecules and death receptor ligands resulted in an increased cytotoxic potential of NK cells, we compared the lysis of target cells by splenic NK cells isolated from FV-infected mice treated with IFN-α11 or left untreated. For the cytotoxicity assay, NK cells were co-cultured with 2 different target cell lines at different effector/target ratios. As positive control, we used YAC-1 cells derived from a Moloney murine leukemia virus-induced lymphoma. These cells have a low H-2 expression, which makes them sensitive to NK cell killing [40]. Furthermore, F-MuLV-induced FBL-3 leukemia cells, a cell type which represents a possible target for NK cells during FV infection *in vivo*, were tested. Fig. 4A shows that NK cells from FV-infected non-treated mice were unable to efficiently lyse the 2 target cell lines. This is in line with our findings that depletion of NK cells in FV-infected non-treated mice did not affect viral loads (Fig. 2B), suggesting that FV infection alone does not efficiently activate NK cells. In contrast, both cell lines were efficiently killed by those NK cells isolated from IFN-α11-treated mice. These data indicate that IFN-α11 treatment strongly improves NK cell cytotoxicity *in vitro*. To determine which cytotoxic pathway was involved in the antiviral activity of IFN-α11-activated NK cells, we tested the cytolytic potential of NK cells in the presence of different inhibitors or neutralizing antibodies. As it is shown in Fig. 4B, the specific lysis of FBL-3 cells was inhibited by the pan-caspase inhibitor Z-VAD-fmk, which was used as a positive control, since it blocks all known cytotoxic pathways of NK cells. In addition, the blockage of the perforin/granzyme pathway by the inhibitor CMA significantly reduced the lysis of FBL-3 cells to less than 45% of control cultures. By contrast, TRAIL or FasL blocking antibodies did not reduce the specific lysis of FBL-3 cells by NK cells from IFN-α11-treated mice. To verify these results, we isolated splenic NK cells from IFN-α11-treated or non-treated FV-infected granzyme B knockout mice (GzmB^−/−^). In contrast to NK cells from wild type mice, NK cells isolated from IFN-α11-treated GzmB^−/−^ mice did not efficiently kill FBL-3 cells (only 29% of the killing from wild type controls) showing that granzyme B was indeed required for the *in vitro* cytotoxicity of activated NK cells (Fig. 4C). To investigate the role of granzyme B during IFN-α11 treatment *in vivo*, we infected wild type mice and GzmB^−/−^ mice with FV and treated them with IFN-α11 (Fig. 4D). At 10 dpi, mice were analyzed for viral loads. In GzmB^−/−^ mice IFN-α11 treatment resulted in a 3.5-fold reduction of the viral loads, whereas in the wild type mice a 20-fold decrease in the viral loads was observed (Fig. 4D). The viral loads in the IFN-α11-treated GzmB^−/−^ mice were similar to those found in the NK cell depletion experiments (Fig. 2B), indicating that IFN-α11 activated NK cells required granzyme B to efficiently target retrovirus-infected cells. The reduction in viral loads in the GzmB^−/−^ mice was probably due to the direct antiviral effect of IFN-α11 (Fig S1).

Apart from a possible direct antiviral effect of IFN-α11-induced ISG, NK cells seem to be the essential factor in the antiviral response against FV induced by IFN-α11. Thus, it should be possible to transfer the therapeutic effect of IFN-α11 by adoptively transferring NK cells from treated mice. To evaluate this, we adoptively transferred 2×10^6^ NK cells from IFN-α11-treated mice into FV-infected recipients. Four days post NK cell transfer, mice were analyzed for viral loads. We observed a 17-fold reduction in FV loads after transfer of NK cells from IFN-α11-treated animals compared to the transfer of NK cells isolated from FV-infected control mice (Fig. 4E). These results further imply that, apart from its direct antiviral effect, IFN-α11 directly and selectively stimulated NK cells during retroviral infection and enabled them to control viral replication.

### IFN-α11 also reduced viral loads in a herpes virus infection

**Figure 5:**
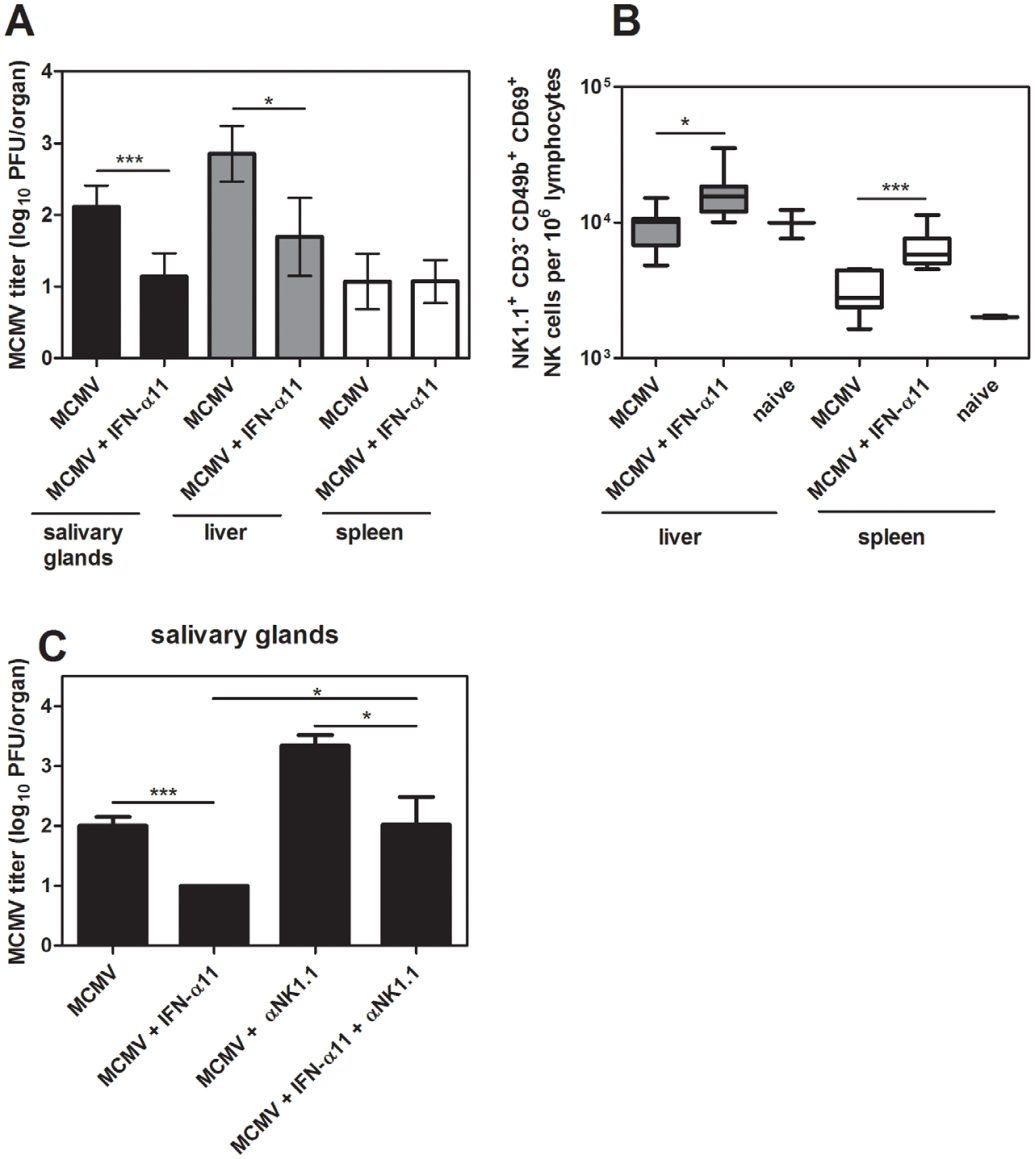
Treatment with IFN-α11 during acute MCMV infection. C57BL/6 mice were treated daily with 8000 units of IFN α11 from −1 through +6 dpi with 5×10^4^ PFU of MCMV. Seven dpi, viral loads in the spleen, liver and salivary glands were analyzed by using a plaque assay (A). NK cells in the liver and spleen were analyzed by flow cytometry. The early activation marker CD69 was used to analyze the frequencies of activated NK cells (NK1.1^+^ CD3^−^ CD49b^+^) (B). C57BL/6 mice were treated daily with 8000 units of IFN-α11 from −1 through +6 dpi with 5×10^4^ PFU of MCMV. NK cells were depleted by 4 injections of supernatant of clone PK136 starting at the day of MCMV infection. IFN-α11-treated non-depleted mice and NK cell depleted, non-treated mice were used as control groups. At 7 dpi, viral loads in the salivary glands were determined by using a plaque assay (C). A minimum of 4 mice per group were analyzed and the mean values are shown by bars. At least 2 independent experiments were performed. Statistically significant differences between the groups are indicated by * for p<0.05, *** for p<0.0005.

As IFN-α11 mediated antiviral activity and improved NK cell responses during FV infection, we investigated whether this is specific for retroviruses or also applicable to other viral infections. Therefore, the antiviral activity of IFN-α11 in a murine CMV (MCMV) infection was analyzed. MCMV is a very interesting model virus for these studies, since NK cells are strongly activated during acute MCMV infection and mediate efficient control of viral replication [31]. We treated MCMV-infected C57BL/6 mice with IFN-α11 according to our standard protocol. Seven dpi, liver, spleen, and salivary glands were analyzed for MCMV loads and NK cell activation. As shown in Fig. 5A, treatment with IFN-α11 during acute MCMV infection significantly reduced viral loads in the salivary glands and liver. Similar to FV infection, we detected a significant increase in NK cell activation in the spleen and liver of MCMV-infected mice after treatment with IFN-α11 (Fig. 5B), demonstrating that even strong antiviral NK cell responses can be further improved by the subtype. To further investigate, if the enhanced NK cell activation mediated by IFN-α11 was required for the elimination of the virus-infected cells, we performed NK cell depletion experiments during MCMV infection. As shown in Fig. 5C, IFN-α11 significantly reduced viral loads in the salivary glands by 60-fold. In contrast, treatment with IFN-α11 in NK cell-depleted mice resulted in only 10-fold reduced viral loads compared to NK cell-depleted controls. These data support our findings in the FV-model that part of the antiviral effect of IFN-α11 was mediated by NK cells whereas the other part depended on the direct antiviral activity of IFN-α11. The results indicate that IFN-α11 is not only effective in retroviral infections but similar mechanisms also apply to herpes virus infections. Thus, IFN-α11 may be an efficient drug for the treatment of many different viral diseases.

## Discussion

As an immediate early response to invading viruses, type I IFN induces an antiviral state in virus-infected and neighboring cells, which is mediated by the expression of antiviral enzymes directly inhibiting viral replication. Type I IFN also modulates the effector function of innate and adaptive immune cells.

In the current study, we have analyzed the role of IFN-α subtypes during the treatment of retroviral infections with a focus on their impact on NK cell responses. Treatment with IFN-α11 inhibited FV-replication in acutely infected mice, whereas IFN-α2 and α5 were not effective. IFN-α11 could on one hand induce antiviral enzymes and on other hand efficiently activate NK cells to kill virus-infected cells. This dual antiviral role of type I IFN has also been suggested to mediate viral clearance during HBV and HCV therapy with human IFN-α2. During the first phase of treatment, a rapid decline in viral loads is observed probably due to the direct antiviral effects induced by IFN-α. In addition, activated NK cells might contribute to this initial control of virus [15]. However, the virus is not completely eliminated during this phase of treatment [43]. In many patients viral loads decrease slowly during the ongoing treatment which can finally result in a total clearance of HBV or HCV. These data suggest that the modulation of innate or adaptive immune cells by IFN-α is required for viral clearance [16], [44], [45]. In the current study we demonstrate for the first time that IFN-α11 acts in such a dual role in a retroviral infection. It induced antiviral enzymes as well as NK cell activation *in vivo* and both mechanisms contributed to the control of virus replication as shown by NK cell depletion (Fig. 2B) and adoptive transfer experiments (Fig. 4E). These experiments demonstrated that about 58% of the antiviral effect was mediated by activated NK cells.

We show here that specifically IFN-α11 stimulates NK cells, resulting in activation and improved effector functions (Fig. 2A and 3), which is similar to the antiviral effects of IFN-α1 on NK cells [32]. IFN-α1 could also enhance the cytotoxic activity against FV-transformed cells and the expression of granzyme B (data not shown), which was not found for the other IFN-α subtypes tested (IFN-α2 and α5; α4, α6 and α9 in [32]). Interestingly, these 2 subtypes share a sequence motif that is different in all other subtypes tested. The specific immunomodulatory activity is not easy to understand, as all IFN-α subtypes bind to the same receptor. One explanation seems to be their different binding affinity to IFNAR1 and 2 which was shown for human IFN-α subtypes [21]. Very similar effects have been observed for chemokines. Various chemokines bind to the same receptor but they all exert different biological functions. Chemokines also differ in their binding profiles to their receptors [46] and this results in differential downstream signaling [47]. It was reported that different IFN-α subtypes induce specific patterns of ISG and can activate different signaling cascades, which might be due to their receptor affinity and the responding cell type [22], [48]–[51]. Various ISG are important for the modulation of immune cell functions, which can explain the different immunomodulatory activities of the different IFN-α subtypes.

In the current study, IFN-α11 improved NK cell responses but not T cell responses. Type I IFN is known to activate NK cells and to further enhance their cytotoxicity [52] but little was known about the different IFN-α subtypes mediating this effect. Three older *in vitro* studies from the 1980s suggest differential effects of human IFN-α subtypes on NK cells [53]–[55] implying that our findings in mouse models may be relevant for humans. Using well-defined mouse models allows to define the basic immunological principles of individual IFN-α subtypes.

In the FV model, virus-infected cells and transformed cells are potential targets for NK cells. However, it was previously shown that NK cells do not play a critical role in immunity during acute FV infection [9], possibly due to very limited IFN-α induction by FV [13], [14]. Others showed that an activation of NK cells prior to FV infection by a vaccination approach resulted in improved control of viral infection [56]. Thus, therapeutic activation of NK cells is required to enable them to control acute FV infection.

In our model, the enhanced killing of virus-infected cells by NK cells was strictly granzyme B dependent (Fig. 4B, C). Earlier studies of our group have established that granzyme A and B as well as perforin plays an important role in cytotoxicity against FV-infected cells [57]. Granzymes are serine proteases that initiate caspase-mediated apoptosis in target cells. Perforin is required to get granzymes into these target cells [58], however also perforin-independent mechanisms of granzyme A and B mediated exocytosis were described [59]. We also detected an increased TRAIL expression on activated NK cells after IFN-α11 treatment. However, in *in vitro* cytotoxicity assays we demonstrated that only the blocking of the granzyme/perforin pathway reduced the cytotoxicity of activated NK cells, whereas anti-TRAIL antibodies did not reduce the killing of FV-transformed cells by NK cells. However, the IFN-α11 induced up-regulation of TRAIL might be important for the treatment of other viral infections or cancer, as numerous studies described TRAIL to be critical for anti-tumor or antiviral NK cell functions [16], [60]–[64].

In retroviral infections such as HIV, no specific NK cell receptors that directly recognize HIV-1-infected cells have been identified up to now [65]. It was suggested that NK cell responses during HIV infection are regulated by a balance of activating and inhibiting signals. For FV infection, one study reported that NKG2D-RAE-1 interaction is critical for the antiviral activity of NK cells [8]. The authors detected an increased expression of RAE-1 on infected erythroblasts, which are the main target cells for FV. Its blockade with anti-RAE-1 antibodies resulted in a reduced NK cell activity against FV-infected cells. NKG2D might also be critical in HIV infection because it was shown that the HIV protein Nef could down-regulate different NKG2D ligands on infected cells, which might contribute to viral escape from NK cell responses [7]. At least in the FV model, IFN-α11 treatment efficiently activated NK cells which might help to overcome such escape mechanisms and enabled NK cells to control viral infection.

It was previously shown that the type I IFN response during acute FV infection is very weak [13], [14]. These results are comparable to those observed for other retroviruses, like HIV or SIV [66]. In HIV and SIV, a transient IFN-α response is found in the early phase of the infection. *In vitro* studies revealed that HIV has multiple factors for suppressing IFN-α responses [67], [68]. It was also shown that during primary HIV infection, type I IFN are barely detectable due to a decrease in plasmacytoid DC numbers [69], the most important IFN-α producers. This was also confirmed in chronic HIV infections [70]. In SIV infections a transient peak of IFN-α was detectable in blood and lymph nodes of rhesus macaques, but the viral loads were not significantly suppressed by these IFN-α levels [71]. Similar to retroviruses, other viruses counteract type I IFN responses by various mechanisms. Thus, the therapeutic treatment of viral infections with exogenous type I IFN, more precisely specific IFN-α subtypes, could have a great potential for future viral immunotherapies.

Our results indicate that distinct IFN-α subtypes have unique biological functions, which have to be characterized in detail to develop such therapies. The usage of specific IFN-α subtypes may also limit their severe side effects that patients currently suffer from during IFN treatment.

## Supporting Information

Figure S1 **Direct antiretroviral activity of IFN-α1** ***1*** **.** (A) *Mus dunni* cells were treated *in vitro* with increasing concentrations of IFN-α2, α5 or α11 (2.5–40 units/ml; A) 24 h prior to infection with 25 FFU/ml F-MuLV. Cells were cultivated for 3 days, fixed with ethanol and stained with F-MuLV envelope-specific antibody 720 to detect foci. The means of 4 independent experiments+SEM are shown. Differences between the untreated control (0) and IFN-α-treated cultures were analyzed. (B) (B10.A×A.BY) F_1_ mice were treated daily with 8000 units of IFN-α11 from −1 through +9 dpi with 7000 SFFU of FV. Ten dpi, splenocytes were isolated from 6 individual mice per group and the total mRNA was isolated using TRIzol. Levels of *Oas1a* and *PKR* mRNA were measured by quantitative RT-PCR. The housekeeping gene *β-actin* was amplified from each sample to normalize the template concentration and used as an internal standard. At least 2 independent experiments were performed and the samples were run in duplicate. Means of six mice per group +SEM are shown. Statistically significant differences between the groups are indicated by *for p<0.05, **for p<0.005 or *** for p<0.0005.
(TIF)

Figure S2 **Analysis of FV-specific T cells from IFN-α11-treated mice.** C57BL/6 mice were treated daily with 8000 units of IFN-α11 from −1 through +9 dpi with 20,000 SFFU of FV. At 10 dpi, spleen cells were analyzed by flow cytometry. The frequencies of virus-specific CD4^+^ (A) and CD8^+^ T cells (B) were analyzed by tetramers. At the same time point, an *in vivo* cytotoxicity assay was performed. Splenocytes from naive mice were loaded with the FV-specific DbGagL CD8^+^ T cell epitope and labeled with CFSE. Target cells were injected i.v. into naive, FV-infected untreated and FV-infected IFN-α11-treated mice. Two hours after transfer, donor cells from spleen were analyzed. The figure shows the percentage of target cell killing in the spleen (C). A minimum of 7 mice in all groups of infected mice were analyzed and the mean value for each group is indicated by a bar. At least 2 independent experiments were performed. Statistically significant differences between the untreated control group (FV) and the IFN-α11-treated mice are indicated by * for p<0.05 or ** for p<0.005.
(TIF)

## References

[1] Trinchieri G (1989) Biology of natural killer cells. Adv Immunol 47: 187–376. 2683611 10.1016/S0065-2776(08)60664-1 PMC7131425

[2] Vivier E, Nunes JA, Vely F (2004) Natural killer cell signaling pathways. Science 306: 1517–1519. 15567854 10.1126/science.1103478

[3] Biron CA, Byron KS, Sullivan JL (1989) Severe herpesvirus infections in an adolescent without natural killer cells. N Engl J Med 320: 1731–1735. 2543925 10.1056/NEJM198906293202605

[4] Vivier E, Tomasello E, Baratin M, Walzer T, Ugolini S (2008) Functions of natural killer cells. Nat Immunol 9: 503–510. 18425107 10.1038/ni1582

[5] Martin MP, Qi Y, Gao X, Yamada E, Martin JN, et al (2007) Innate partnership of HLA-B and KIR3DL1 subtypes against HIV-1. Nat Genet 39: 733–740. 17496894 10.1038/ng2035 PMC4135476

[6] Fellay J, Shianna KV, Ge D, Colombo S, Ledergerber B, et al (2007) A whole-genome association study of major determinants for host control of HIV-1. Science 317: 944–947. 17641165 10.1126/science.1143767 PMC1991296

[7] Cerboni C, Neri F, Casartelli N, Zingoni A, Cosman D, et al (2007) Human immunodeficiency virus 1 Nef protein downmodulates the ligands of the activating receptor NKG2D and inhibits natural killer cell-mediated cytotoxicity. J Gen Virol 88: 242–250. 17170457 10.1099/vir.0.82125-0

[8] Ogawa T, Tsuji-Kawahara S, Yuasa T, Kinoshita S, Chikaishi T, et al (2011) Natural killer cells recognize friend retrovirus-infected erythroid progenitor cells through NKG2D-RAE-1 interactions In Vivo. J Virol 85: 5423–5435. 21411527 10.1128/JVI.02146-10 PMC3094956

[9] Zelinskyy G, Balkow S, Schimmer S, Werner T, Simon MM, et al (2007) The level of friend retrovirus replication determines the cytolytic pathway of CD8+ T-cell-mediated pathogen control. J Virol 81: 11881–11890. 17728236 10.1128/JVI.01554-07 PMC2168789

[10] Ferlazzo G, Pack M, Thomas D, Paludan C, Schmid D, et al (2004) Distinct roles of IL-12 and IL-15 in human natural killer cell activation by dendritic cells from secondary lymphoid organs. Proc Natl Acad Sci U S A 101: 16606–16611. 15536127 10.1073/pnas.0407522101 PMC534504

[11] Trinchieri G, Santoli D, Granato D, Perussia B (1981) Antagonistic effects of interferons on the cytotoxicity mediated by natural killer cells. Fed Proc 40: 2705–2710. 6169557

[12] Stacey AR, Norris PJ, Qin L, Haygreen EA, Taylor E, et al (2009) Induction of a striking systemic cytokine cascade prior to peak viremia in acute human immunodeficiency virus type 1 infection, in contrast to more modest and delayed responses in acute hepatitis B and C virus infections. J Virol 83: 3719–3733. 19176632 10.1128/JVI.01844-08 PMC2663284

[13] Gerlach N, Schimmer S, Weiss S, Kalinke U, Dittmer U (2007) Effects of Type I Interferons on Friend Retrovirus Infection (Erratum). J Virol 81: 6160. 10.1128/JVI.80.7.3438-3444.2006 PMC1440373 16537611

[14] Gerlach N, Schimmer S, Weiss S, Kalinke U, Dittmer U (2006) Effects of type I interferons on Friend retrovirus infection. J Virol 80: 3438–3444. 16537611 10.1128/JVI.80.7.3438-3444.2006 PMC1440373

[15] Ahlenstiel G, Edlich B, Hogdal LJ, Rotman Y, Noureddin M, et al (2011) Early changes in natural killer cell function indicate virologic response to interferon therapy for hepatitis C. Gastroenterology. 141: 1231–1239. 10.1053/j.gastro.2011.06.069 PMC3353552 21741920

[16] Stegmann KA, Bjorkstrom NK, Veber H, Ciesek S, Riese P, et al (2010) Interferon-alpha-induced TRAIL on natural killer cells is associated with control of hepatitis C virus infection. Gastroenterology 138: 1885–1897. 20334827 10.1053/j.gastro.2010.01.051

[17] Miyazawa M, Nishio J, Chesebro B (1992) Protection against Friend retrovirus-induced leukemia by recombinant vaccinia viruses expressing the gag gene. J Virol 66: 4497–4507. 1534853 10.1128/jvi.66.7.4497-4507.1992 PMC241259

[18] van Pesch V, Lanaya H, Renauld JC, Michiels T (2004) Characterization of the murine alpha interferon gene family. J Virol 78: 8219–8228. 15254193 10.1128/JVI.78.15.8219-8228.2004 PMC446145

[19] Hardy MP, Owczarek CM, Jermiin LS, Ejdeback M, Hertzog PJ (2004) Characterization of the type I interferon locus and identification of novel genes. Genomics 84: 331–345. 15233997 10.1016/j.ygeno.2004.03.003

[20] Aguet M, Grobke M, Dreiding P (1984) Various human interferon alpha subclasses cross-react with common receptors: their binding affinities correlate with their specific biological activities. Virology 132: 211–216. 6320534 10.1016/0042-6822(84)90105-3

[21] Jaks E, Gavutis M, Uze G, Martal J, Piehler J (2007) Differential receptor subunit affinities of type I interferons govern differential signal activation. J Mol Biol 366: 525–539. 17174979 10.1016/j.jmb.2006.11.053

[22] Cull VS, Tilbrook PA, Bartlett EJ, Brekalo NL, James CM (2003) Type I interferon differential therapy for erythroleukemia: specificity of STAT activation. Blood 101: 2727–2735. 12446459 10.1182/blood-2002-05-1521

[23] Moll HP, Maier T, Zommer A, Lavoie T, Brostjan C (2011) The differential activity of interferon-alpha subtypes is consistent among distinct target genes and cell types. Cytokine 53: 52–59. 20943413 10.1016/j.cyto.2010.09.006 PMC3020287

[24] Austin BA, James C, Silverman RH, Carr DJ (2005) Critical role for the oligoadenylate synthetase/RNase L pathway in response to IFN-beta during acute ocular herpes simplex virus type 1 infection. J Immunol 175: 1100–1106. 16002711 10.4049/jimmunol.175.2.1100

[25] Harle P, Cull V, Agbaga MP, Silverman R, Williams BR, et al (2002) Differential effect of murine alpha/beta interferon transgenes on antagonization of herpes simplex virus type 1 replication. J Virol 76: 6558–6567. 12050368 10.1128/JVI.76.13.6558-6567.2002 PMC136290

[26] James CM, Abdad MY, Mansfield JP, Jacobsen HK, Vind AR, et al (2007) Differential activities of alpha/beta IFN subtypes against influenza virus in vivo and enhancement of specific immune responses in DNA vaccinated mice expressing haemagglutinin and nucleoprotein. Vaccine 25: 1856–1867. 17240000 10.1016/j.vaccine.2006.10.038

[27] Yeow WS, Lai CM, Beilharz MW (1997) The in vivo expression patterns of individual type I interferon genes in murine cytomegalovirus infections. Antiviral Res 34: 17–26. 9107382 10.1016/s0166-3542(96)01018-2

[28] Muller U, Steinhoff U, Reis LF, Hemmi S, Pavlovic J, et al (1994) Functional role of type I and type II interferons in antiviral defense. Science 264: 1918–1921. 8009221 10.1126/science.8009221

[29] Heusel JW, Wesselschmidt RL, Shresta S, Russell JH, Ley TJ (1994) Cytotoxic lymphocytes require granzyme B for the rapid induction of DNA fragmentation and apoptosis in allogeneic target cells. Cell 76: 977–987. 8137431 10.1016/0092-8674(94)90376-x

[30] Robertson SJ, Ammann CG, Messer RJ, Carmody AB, Myers L, et al (2008) Suppression of acute anti-friend virus CD8+ T-cell responses by coinfection with lactate dehydrogenase-elevating virus. J Virol 82: 408–418. 17959678 10.1128/JVI.01413-07 PMC2224392

[31] Bukowski JF, Woda BA, Habu S, Okumura K, Welsh RM (1983) Natural killer cell depletion enhances virus synthesis and virus-induced hepatitis in vivo. J Immunol 131: 1531–1538. 6309965

[32] Gerlach N, Gibbert K, Alter C, Nair S, Zelinskyy G, et al (2009) Anti-retroviral effects of type I IFN subtypes in vivo. Eur J Immunol 39: 136–146. 19130550 10.1002/eji.200838311

[33] Bollati-Fogolin M, Muller W (2005) Virus free, cell-based assay for the quantification of murine type I interferons. J Immunol Methods 306: 169–175. 16209875 10.1016/j.jim.2005.08.005

[34] Dittmer U, Brooks DM, Hasenkrug KJ (1998) Characterization of a live-attenuated retroviral vaccine demonstrates protection via immune mechanisms. J Virol 72: 6554–6558. 9658099 10.1128/jvi.72.8.6554-6558.1998 PMC109828

[35] Chen HD, Fraire AE, Joris I, Welsh RM, Selin LK (2003) Specific history of heterologous virus infections determines anti-viral immunity and immunopathology in the lung. Am J Pathol 163: 1341–1355. 14507643 10.1016/S0002-9440(10)63493-1 PMC1868309

[36] Chen W, Qin H, Chesebro B, Cheever MA (1996) Identification of a gag-encoded cytotoxic T-lymphocyte epitope from FBL-3 leukemia shared by Friend, Moloney, and Rauscher murine leukemia virus-induced tumors. J Virol 70: 7773–7782. 8892898 10.1128/jvi.70.11.7773-7782.1996 PMC190847

[37] Shimizu T, Uenishi H, Teramura Y, Iwashiro M, Kuribayashi K, et al (1994) Fine structure of a virus-encoded helper T-cell epitope expressed on FBL-3 tumor cells. J Virol 68: 7704–7708. 7525983 10.1128/jvi.68.12.7704-7708.1994 PMC237231

[38] Zelinskyy G, Kraft AR, Schimmer S, Arndt T, Dittmer U (2006) Kinetics of CD8+ effector T cell responses and induced CD4+ regulatory T cell responses during Friend retrovirus infection. Eur J Immunol 36: 2658–2670. 16981182 10.1002/eji.200636059

[39] Zelinskyy G, Dietze KK, Husecken YP, Schimmer S, Nair S, et al (2009) The regulatory T-cell response during acute retroviral infection is locally defined and controls the magnitude and duration of the virus-specific cytotoxic T-cell response. Blood 114: 3199–3207. 19671923 10.1182/blood-2009-03-208736

[40] Cikes M, Friberg S Jr, Klein G (1973) Progressive loss of H-2 antigens with concomitant increase of cell-surface antigen(s) determined by Moloney leukemia virus in cultured murine lymphomas. J Natl Cancer Inst 50: 347–362. 4573851 10.1093/jnci/50.2.347

[41] McCoy JL, Fefer A, Glynn JP (1967) Influence of infectious virus on the induction of transplantation resistance in the Friend tumor system. Cancer Res 27: 2267–2271. 4866760

[42] Kataoka T, Shinohara N, Takayama H, Takaku K, Kondo S, et al (1996) Concanamycin A, a powerful tool for characterization and estimation of contribution of perforin- and Fas-based lytic pathways in cell-mediated cytotoxicity. J Immunol 156: 3678–3686. 8621902

[43] Neumann AU, Lam NP, Dahari H, Gretch DR, Wiley TE, et al (1998) Hepatitis C viral dynamics in vivo and the antiviral efficacy of interferon-alpha therapy. Science 282: 103–107. 9756471 10.1126/science.282.5386.103

[44] Feld JJ, Hoofnagle JH (2005) Mechanism of action of interferon and ribavirin in treatment of hepatitis C. Nature 436: 967–972. 16107837 10.1038/nature04082

[45] Herrmann E, Lee JH, Marinos G, Modi M, Zeuzem S (2003) Effect of ribavirin on hepatitis C viral kinetics in patients treated with pegylated interferon. Hepatology 37: 1351–1358. 12774014 10.1053/jhep.2003.50218

[46] Graham GJ (2009) D6 and the atypical chemokine receptor family: novel regulators of immune and inflammatory processes. Eur J Immunol 39: 342–351. 19130487 10.1002/eji.200838858

[47] Zidar DA, Violin JD, Whalen EJ, Lefkowitz RJ (2009) Selective engagement of G protein coupled receptor kinases (GRKs) encodes distinct functions of biased ligands. Proc Natl Acad Sci U S A 106: 9649–9654. 19497875 10.1073/pnas.0904361106 PMC2689814

[48] Grumbach IM, Fish EN, Uddin S, Majchrzak B, Colamonici OR, et al (1999) Activation of the Jak-Stat pathway in cells that exhibit selective sensitivity to the antiviral effects of IFN-beta compared with IFN-alpha. J Interferon Cytokine Res 19: 797–801. 10454351 10.1089/107999099313659

[49] Der SD, Zhou A, Williams BR, Silverman RH (1998) Identification of genes differentially regulated by interferon alpha, beta, or gamma using oligonucleotide arrays. Proc Natl Acad Sci U S A 95: 15623–15628. 9861020 10.1073/pnas.95.26.15623 PMC28094

[50] Leaman DW, Chawla-Sarkar M, Jacobs B, Vyas K, Sun Y, et al (2003) Novel growth and death related interferon-stimulated genes (ISGs) in melanoma: greater potency of IFN-beta compared with IFN-alpha2. J Interferon Cytokine Res 23: 745–756. 14769151 10.1089/107999003772084860

[51] Severa M, Remoli ME, Giacomini E, Ragimbeau J, Lande R, et al (2006) Differential responsiveness to IFN-alpha and IFN-beta of human mature DC through modulation of IFNAR expression. J Leukoc Biol 79: 1286–1294. 16624932 10.1189/jlb.1205742

[52] Biron CA, Nguyen KB, Pien GC, Cousens LP, Salazar-Mather TP (1999) Natural killer cells in antiviral defense: function and regulation by innate cytokines. Annu Rev Immunol 17: 189–220. 10358757 10.1146/annurev.immunol.17.1.189

[53] Edwards BS, Hawkins MJ, Borden EC (1984) Comparative in vivo and in vitro activation of human natural killer cells by two recombinant alpha-interferons differing in antiviral activity. Cancer Res 44: 3135–3139. 6586292

[54] Ortaldo JR, Herberman RB, Harvey C, Osheroff P, Pan YC, et al (1984) A species of human alpha interferon that lacks the ability to boost human natural killer activity. Proc Natl Acad Sci U S A 81: 4926–4929. 6589637 10.1073/pnas.81.15.4926 PMC391605

[55] Verhagen A, Mackay IR, Rowley M, Tymms M (1990) Comparison of augmentation of human natural killer cell cytotoxicity by interferon-alpha subtypes. Nat Immun Cell Growth Regul 9: 325–333. 2077397

[56] Iwanami N, Niwa A, Yasutomi Y, Tabata N, Miyazawa M (2001) Role of natural killer cells in resistance against friend retrovirus-induced leukemia. J Virol 75: 3152–3163. 11238842 10.1128/JVI.75.7.3152-3163.2001 PMC114109

[57] Zelinskyy G, Balkow S, Schimmer S, Schepers K, Simon MM, et al (2004) Independent roles of perforin, granzymes, and Fas in the control of Friend retrovirus infection. Virology 330: 365–374. 15567431 10.1016/j.virol.2004.08.040

[58] Jans DA, Jans P, Briggs LJ, Sutton V, Trapani JA (1996) Nuclear transport of granzyme B (fragmentin-2). Dependence of perforin in vivo and cytosolic factors in vitro. J Biol Chem 271: 30781–30789. 8940058 10.1074/jbc.271.48.30781

[59] Simon HG, Fruth U, Kramer MD, Simon MM (1987) A secretable serine proteinase with highly restricted specificity from cytolytic T lymphocytes inactivates retrovirus-associated reverse transcriptase. FEBS Lett 223: 352–360. 2444461 10.1016/0014-5793(87)80318-6

[60] Sato K, Hida S, Takayanagi H, Yokochi T, Kayagaki N, et al (2001) Antiviral response by natural killer cells through TRAIL gene induction by IFN-alpha/beta. Eur J Immunol 31: 3138–3146. 11745330 10.1002/1521-4141(200111)31:11<3138::aid-immu3138>3.0.co;2-b

[61] Kayagaki N, Yamaguchi N, Nakayama M, Eto H, Okumura K, et al (1999) Type I interferons (IFNs) regulate tumor necrosis factor-related apoptosis-inducing ligand (TRAIL) expression on human T cells: A novel mechanism for the antitumor effects of type I IFNs. J Exp Med 189: 1451–1460. 10224285 10.1084/jem.189.9.1451 PMC2193058

[62] Kayagaki N, Yamaguchi N, Nakayama M, Takeda K, Akiba H, et al (1999) Expression and function of TNF-related apoptosis-inducing ligand on murine activated NK cells. J Immunol 163: 1906–1913. 10438925

[63] Walczak H, Miller RE, Ariail K, Gliniak B, Griffith TS, et al (1999) Tumoricidal activity of tumor necrosis factor-related apoptosis-inducing ligand in vivo. Nat Med 5: 157–163. 9930862 10.1038/5517

[64] Zamai L, Ahmad M, Bennett IM, Azzoni L, Alnemri ES, et al (1998) Natural killer (NK) cell-mediated cytotoxicity: differential use of TRAIL and Fas ligand by immature and mature primary human NK cells. J Exp Med 188: 2375–2380. 9858524 10.1084/jem.188.12.2375 PMC2212426

[65] Altfeld M, Fadda L, Frleta D, Bhardwaj N (2011) DCs and NK cells: critical effectors in the immune response to HIV-1. Nat Rev Immunol 11: 176–186. 21350578 10.1038/nri2935 PMC3278081

[66] Hosmalin A, Lebon P (2006) Type I interferon production in HIV-infected patients. J Leukoc Biol 80: 984–993. 16916960 10.1189/jlb.0306154

[67] Harman AN, Lai J, Turville S, Samarajiwa S, Gray L, et al (2011) HIV infection of dendritic cells subverts the IFN induction pathway via IRF-1 and inhibits type 1 IFN production. Blood 118: 298–308. 21411754 10.1182/blood-2010-07-297721 PMC4123420

[68] Yan N, Regalado-Magdos AD, Stiggelbout B, Lee-Kirsch MA, Lieberman J (2010) The cytosolic exonuclease TREX1 inhibits the innate immune response to human immunodeficiency virus type 1. Nat Immunol 11: 1005–1013. 20871604 10.1038/ni.1941 PMC2958248

[69] Kamga I, Kahi S, Develioglu L, Lichtner M, Maranon C, et al (2005) Type I interferon production is profoundly and transiently impaired in primary HIV-1 infection. J Infect Dis 192: 303–310. 15962225 10.1086/430931

[70] Feldman S, Stein D, Amrute S, Denny T, Garcia Z, et al (2001) Decreased interferon-alpha production in HIV-infected patients correlates with numerical and functional deficiencies in circulating type 2 dendritic cell precursors. Clin Immunol 101: 201–210. 11683579 10.1006/clim.2001.5111

[71] Khatissian E, Tovey MG, Cumont MC, Monceaux V, Lebon P, et al (1996) The relationship between the interferon alpha response and viral burden in primary SIV infection. AIDS Res Hum Retroviruses 12: 1273–1278. 8870849 10.1089/aid.1996.12.1273

