# Supplementary figures and images for: Interferon-alpha Subtype 11 Activates NK Cells and Enables Control of Retroviral Infection
Source: PLoS Pathog. 2012 Aug 9;8(8):e1002868. doi: 10.1371/journal.ppat.1002868 (PMC3415439; doi:10.1371/journal.ppat.1002868)

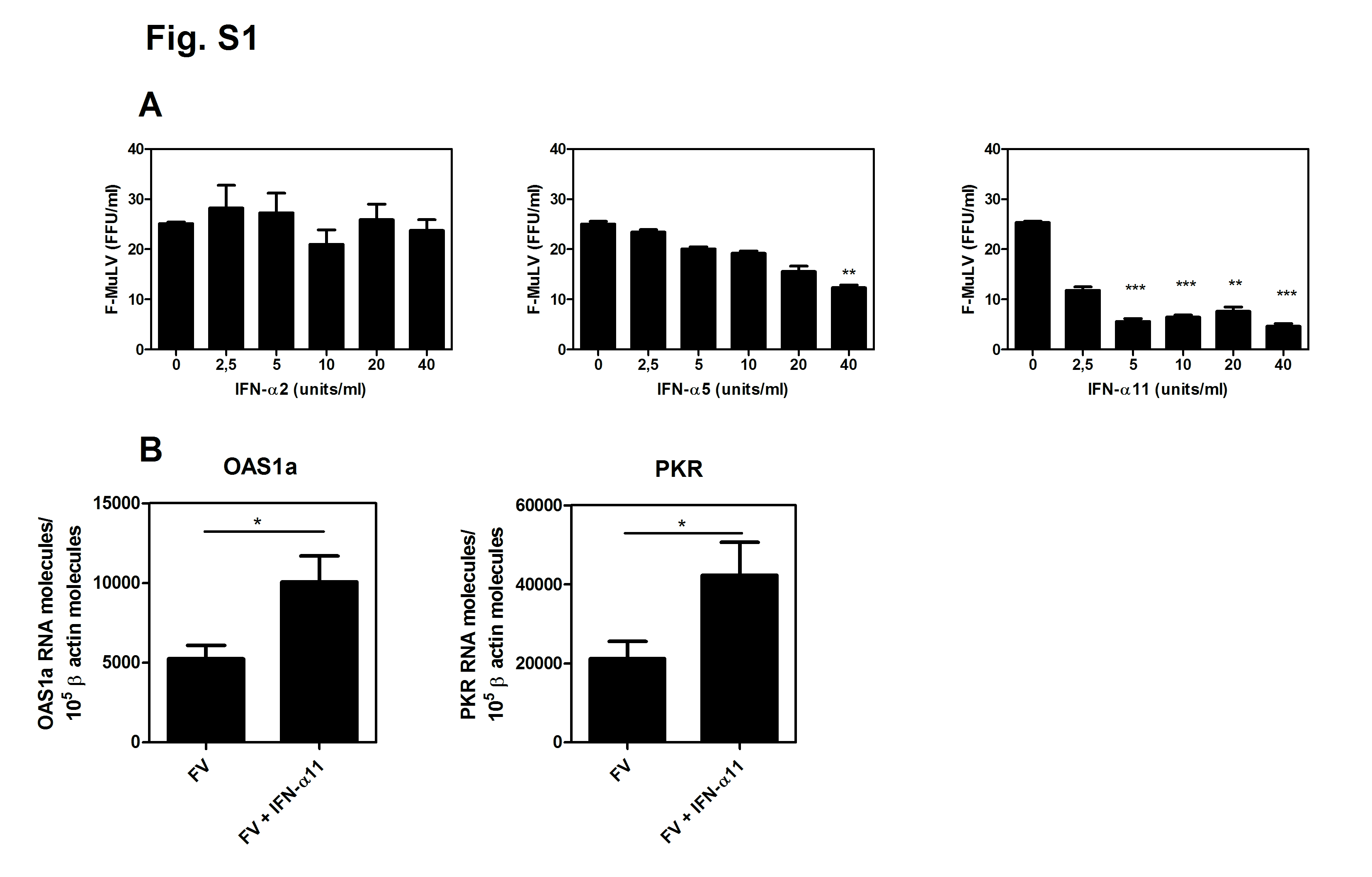

Supplement: Figure S1 — Direct antiretroviral activity of IFN-α1 1 . (A) Mus dunni cells were treated in vitro with increasing concentrations of IFN-α2, α5 or α11 (2.5–40 units/ml; A) 24 h prior to infection with 25 FFU/ml F-MuLV. Cells were cultivated for 3 days, fixed with ethanol and stained with F-MuLV envelope-specific antibody 720 to detect foci. The means of 4 independent experiments+SEM are shown. Differences between the untreated control (0) and IFN-α-treated cultures were analyzed. (B) (B10.A×A.BY) F1 mice were treated daily with 8000 units of IFN-α11 from −1 through +9 dpi with 7000 SFFU of FV. Ten dpi, splenocytes were isolated from 6 individual mice per group and the total mRNA was isolated using TRIzol. Levels of Oas1a and PKR mRNA were measured by quantitative RT-PCR. The housekeeping gene β-actin was amplified from each sample to normalize the template concentration and used as an internal standard. At least 2 independent experiments were performed and the samples were run in duplicate. Means of six mice per group +SEM are shown. Statistically significant differences between the groups are indicated by *for p<0.05, **for p<0.005 or *** for p<0.0005. (TIF) [file ppat.1002868.s001.tif]

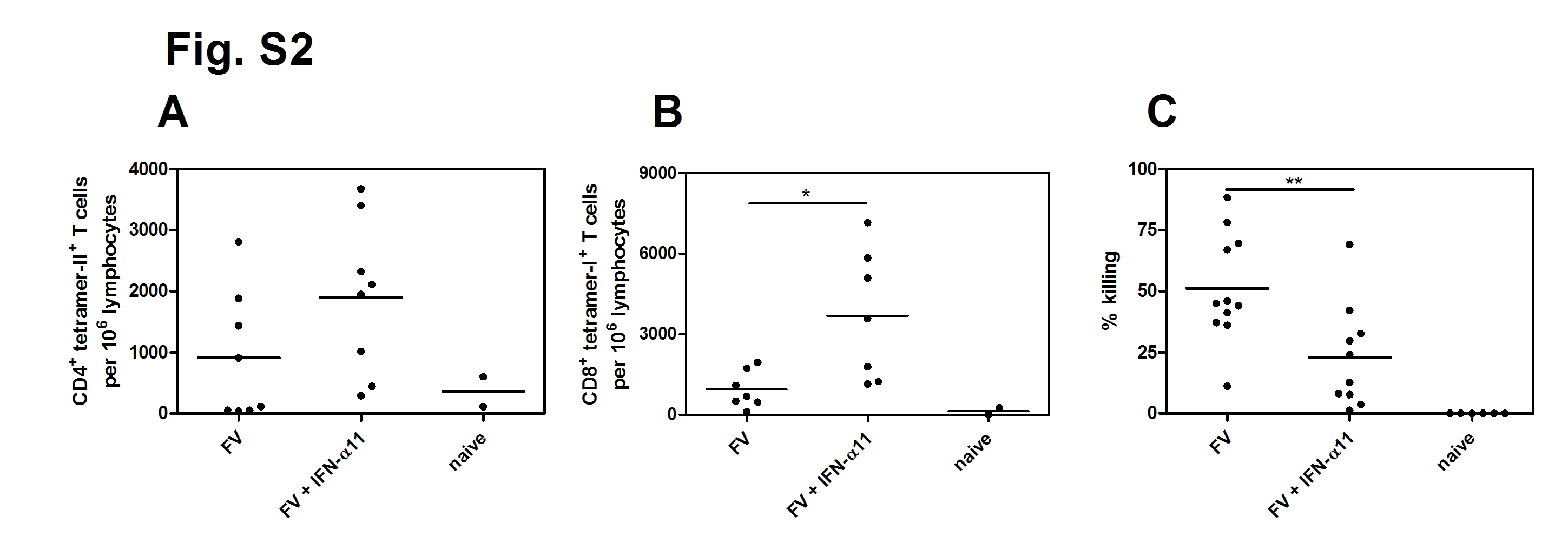

Supplement: Figure S2 — Analysis of FV-specific T cells from IFN-α11-treated mice. C57BL/6 mice were treated daily with 8000 units of IFN-α11 from −1 through +9 dpi with 20,000 SFFU of FV. At 10 dpi, spleen cells were analyzed by flow cytometry. The frequencies of virus-specific CD4+ (A) and CD8+ T cells (B) were analyzed by tetramers. At the same time point, an in vivo cytotoxicity assay was performed. Splenocytes from naive mice were loaded with the FV-specific DbGagL CD8+ T cell epitope and labeled with CFSE. Target cells were injected i.v. into naive, FV-infected untreated and FV-infected IFN-α11-treated mice. Two hours after transfer, donor cells from spleen were analyzed. The figure shows the percentage of target cell killing in the spleen (C). A minimum of 7 mice in all groups of infected mice were analyzed and the mean value for each group is indicated by a bar. At least 2 independent experiments were performed. Statistically significant differences between the untreated control group (FV) and the IFN-α11-treated mice are indicated by * for p<0.05 or ** for p<0.005. (TIF) [file ppat.1002868.s002.tif]
